# Fully phased genome assemblies and graph-based genetic variants of the olive flounder, *Paralichthys olivaceus*

**DOI:** 10.1038/s41597-024-04033-1

**Published:** 2024-11-04

**Authors:** Julan Kim, Yoonsik Kim, Jeongwoen Shin, Yeong-Kuk Kim, Doo Ho Lee, Jong-Won Park, Dain Lee, Hyun-Chul Kim, Jeong-Ho Lee, Seung Hwan Lee, Jun Kim

**Affiliations:** 1 Genetics and Breeding Research Center, National Institute of Fisheries Science, Geoje, 53334 Korea; 2 Department of Bio-AI Convergence, Chungnam National University, Daejeon, 34134 Korea; 3 Quantomic research and solution, Yuseong-gu Daejeon Tips-town, Daejeon, 34134 Korea; 4 Division of Animal & Dairy Science, Chungnam National University, Daejeon, 34134 Korea; 5 Research and Development Planning and Coordination Department, National Institute of Fisheries Science, Busan, 46083 Korea; 6 Department of Convergent Bioscience and Informatics, Chungnam National University, Daejeon, 34134 Korea

**Keywords:** Animal breeding, DNA sequencing, Comparative genomics, Structural variation

## Abstract

The olive flounder, *Paralichthys olivaceus*, also known as the Korean halibut, is an economically important flatfish in East Asian countries. Here, we provided four fully phased genome assemblies of two different olive flounder individuals using high-fidelity long-read sequencing and their parental short-read sequencing data. We obtained 42–44 Gb of ~15-kb and ~Q30 high-fidelity long reads, and their assembly quality values were ~53. We annotated ~30 K genes, ~170-Mb repetitive sequences, and ~3 M 5-methylcytosine positions for each genome assembly, and established a graph-based draft pan-genome of the olive flounder. We identified 5 M single-nucleotide variants and 100 K structural variants with their genotype information, where ~13% of the variants were possibly fixed in the two Korean individuals. Based on our chromosome-level genome assembly, we also explored chromosome evolution in the Pleuronectiformes family, as reported earlier. Our high-quality genomic resources will contribute to future genomic selection for accelerating the breeding process of the olive flounder.

## Background & Summary

The olive flounder (*Paralichthys olivaceus*) is one of the key aquatic resources in the East Asian region, known for its high yield and fast growth rate, among other characteristics^1^. It has been cultured in South Korea since the late 1990s, and during this period, various aspects including feed and rearing conditions have been improved, leading to increased productivity^2–5^. Its aquaculture production had sharply increased over 2000% between 2003 and 2013, and now it holds the largest portion among cultured fishes in South Korea^6^. To meet the high demand for this species and ensure its sustainability, genomic breeding for growth rate and disease resistance has been actively conducted for its genetic improvement^6,7^.

Indeed, genetic and genomic resources of the olive flounder have been accumulated for genomic breeding, such as chromosome-level genomes, linkage maps, transcriptomes, and genome-wide single-nucleotide variants (SNVs)^8–12^. However, these plentiful resources had mainly been produced using conventional short-read sequencing technologies and any high-quality genome is not available for Korean individuals of the olive flounder, lacking sufficient resolution for genetically complex and divergent genomic regions. Many of these resources could be further improved using recently advanced long-read sequencing technologies that can provide high-quality genomic and transcriptomic information to tackle such complex variants^13–20^.

Recently, a Chinese flounder genome based on high-fidelity (HiFi) long-read sequencing was reported, (hereafter, Xu genome) partially alleviating some of the limitations^21^. However, the published genome still comes with several constraints: First, the genome was sequenced before a deep learning-based model enhances the accuracy of HiFi reads^22^, and only HiFi reads were obtained to assemble the genome without short-read sequencing data that can be utilized to estimate base-level accuracy of the genome assembly. Moreover, the flounder genome presented only a primary haplotig assembly of an individual rather than two partially phased assemblies that can be solely constructed using HiFi reads^23,24^. Since it contains only one haplotype assembly and no other long-read-based genome assembly is available, it is difficult to understand large genetic variants and their genotype information in the population^21^. All of this information can constitute pivotal cornerstones for enhancing breeding procedures of this species through genomic selection, which requires producing complete sets of sequencing data.

**Fig. 1:**
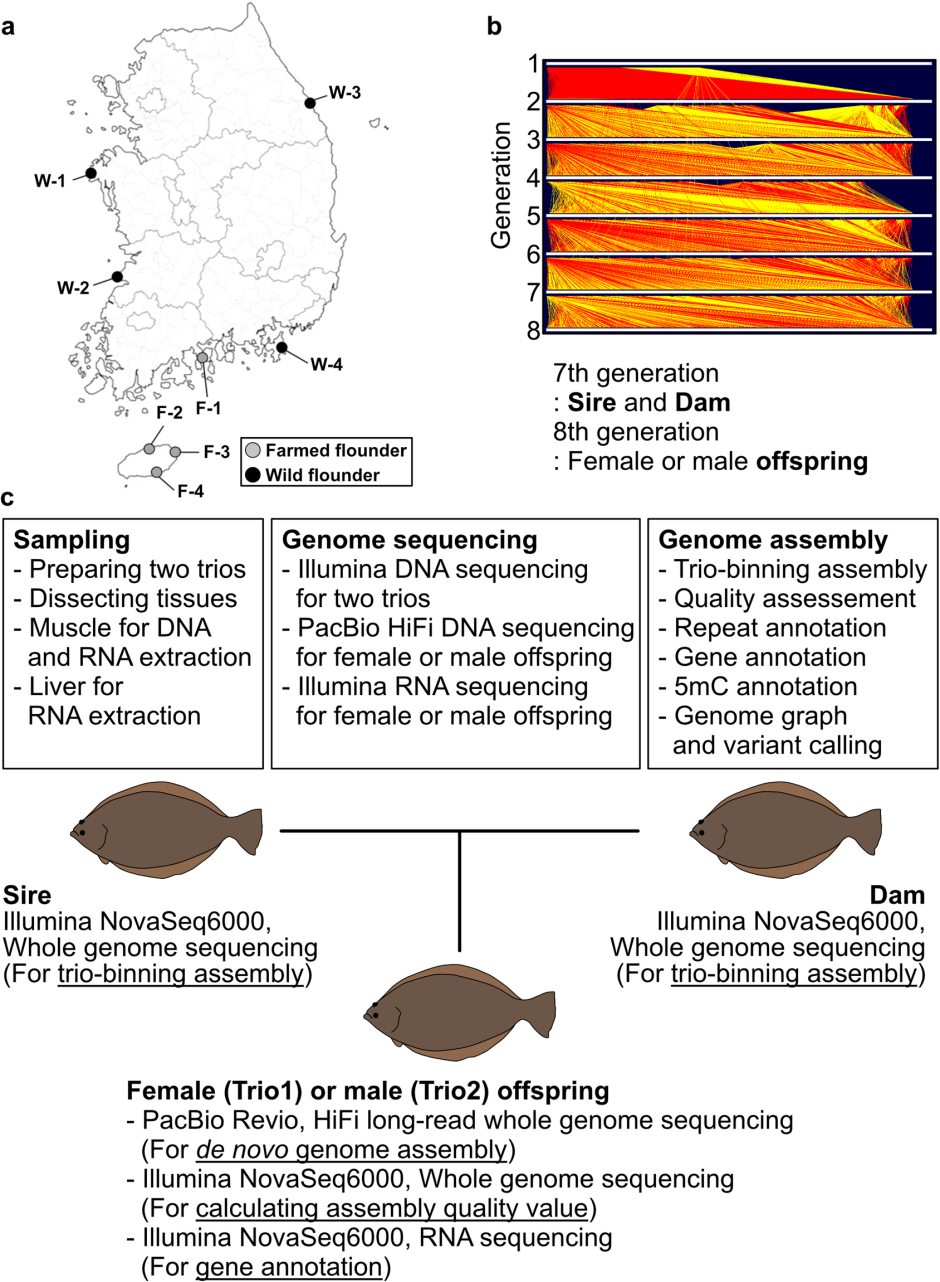
Experimental scheme for this study. (**a**) Sampling locations of the initial flounders for genomic breeding. (**b**) Pedigree information of 54,159 samples in 8 generations. Red lines represent sire-offspring relationship and yellow lines represent dam-offspring relationship. (**c**) Experimental summary for sampling, sequencing, and genome assembly processes.

To overcome these limitations, we provide four fully phased genome assemblies of the olive flounder using two trios (Trio1, sire-dam-female offspring; Trio2, sire-dam-male offspring; Fig. 1). We first obtained HiFi long-read sequencing data of the two offspring and short-read DNA sequencing data of all the six individuals in addition to short-read RNA sequencing data of the two offspring individuals. These HiFi reads were *de novo* assembled into the four fully phased genome assemblies using their parental short-read DNA sequencing data, of which have comparable assembly quality to the previous Xu genome. We measured base-level quality of the assemblies using their own short-read DNA sequencing data and annotated repetitive sequences, genic regions, 5-methylcytosine sites, and genetic variants that were constructed by a graph-based pangenome analysis pipeline. Our chromosome-level genome assembly recapitulated well-known Robertsonian fusions in the Pleuronectiformes family. Our high-quality genomic resources of the olive flounder will enable future advances of their genomic breeding.

## Methods

### Fish source

The flounder used in this study were bred at the facility of Fish Genetics and Breeding Research Centre, South Korea. A total of 1,876 olive flounder were initially chosen as the breeding population in 2003–2004, sourced from four wild and four farm populations (Fig. 1a). The geographic locations and nomenclature are illustrated in Fig. 1a. The larvae were raised in circular fiberglass tanks measuring 150 cm in diameter, 100 cm in height, with a water volume of approximately 1,000 L. These tanks were supplied with filtered seawater and underwent ultraviolet (UV) light treatment. Rotifers were used as the primary feed for the fish from 3 to 20 days after hatching, followed by *Artemia* until 31 days post-hatching, and extruded pellets until harvest. Throughout the production period, water temperatures ranged from 13.7 to 27.3 °C, salinity levels ranged from 30.7 to 34.8 psu, and dissolved oxygen levels ranged from 7.0 to 11.5 mg/L. For artificial fertilization, the parentage assignment was estimated by eight microsatellite loci. Based on these loci, a breeding guideline was prepared to ensure genetic diversity, resulting in the production of more than 200 families in each generation (Fig. 1b). With this process, we have conducted selective breeding of the olive flounder for up to 8 generations. The offspring individuals used in this analysis were randomly selected from the 8th generation.

### Data collection and sequencing

**Fig. 2:**
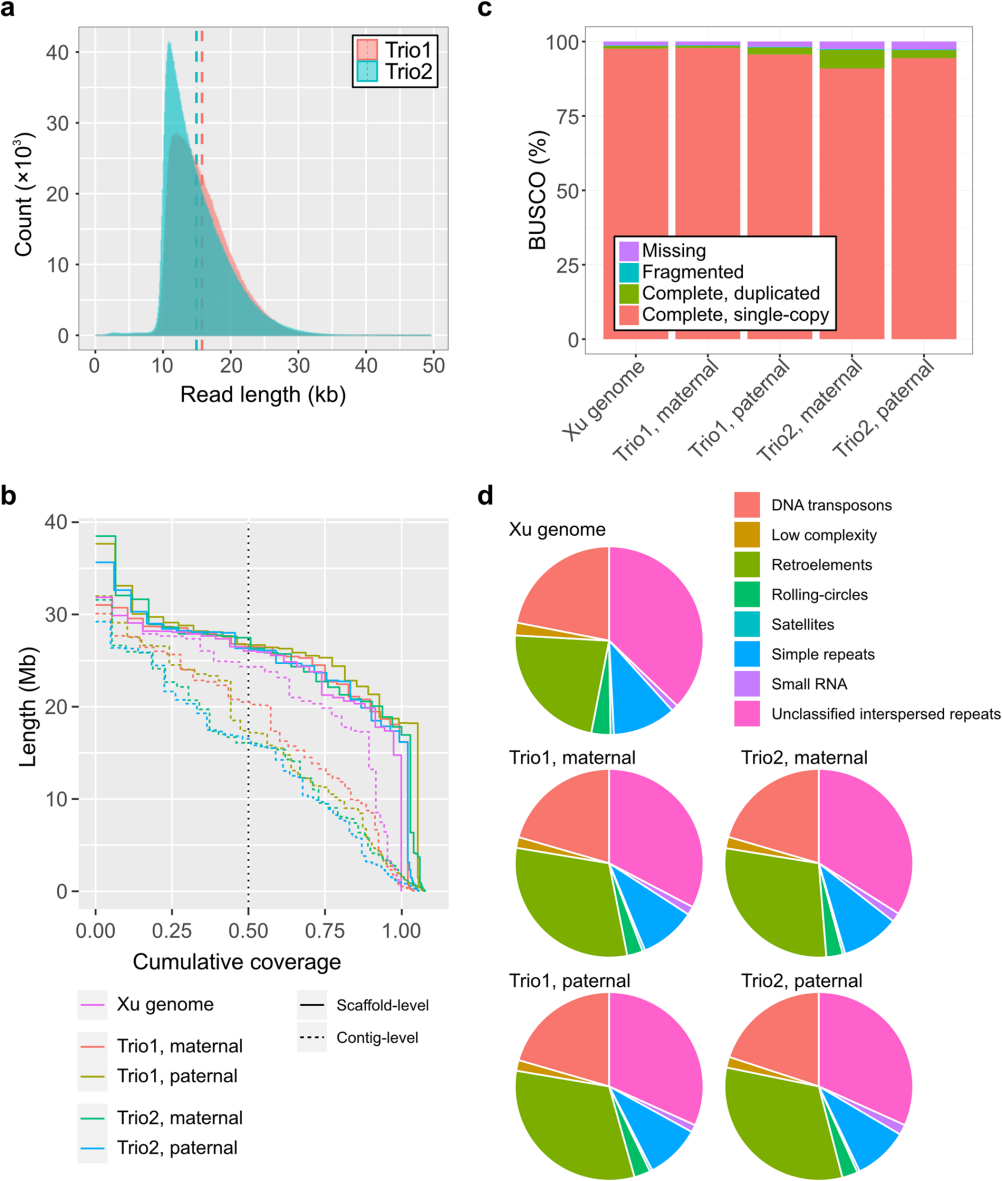
Assembly quality metrics. (**a**) HiFi read length distributions for female offspring of Trio1 and male offspring of Trio2. (**b**) NG50 plot for the Xu genome and our four fully phased genome assemblies. Dotted lines represent contig length distributions and solid lines represent chromosome-level scaffold length distributions. The vertical dotted black line represents NG50. The Xu genome was used for calculating the genome size of this species. (**c**) BUSCO values of the Xu genome and our four genome assemblies. (**d**) Compositions of repetitive sequences in the Xu genome and our genome assemblies.

A total of two trios, consisting of the sire, dam, and female (Trio1) or male (Trio2) offspring, were selected from the 7th and 8th generation for sequencing (Fig. 1b,c). Their muscle tissues were utilized for DNA sequencing and muscle and liver tissues were for RNA sequencing. We produced HiFi reads of two individuals of *P*. *olivaceus* using the PacBio Revio system and Illumina reads of their parents using the Illumina NovaSeq 6000 platform with the TruSeq DNA Nano 550 bp library kit. In addition, muscle and liver tissues of the two individuals were processed, and RNA-seq data were generated using the Illumina NovaSeq 6000 platform with the TruSeq Stranded mRNA library kit. All the DNA and RNA extraction and sequencing were conducted by DNALINK (http://en.dnalink.com/?redirect = no). We produced 41.6 Gb (71×) and 43.5 Gb (74×) of HiFi reads, 25.4–29.5 Gb of Illumina DNA reads (43–50× compared to the chromosome-level Xu genome), and 61M–91 M Illumina RNA reads^25^ (Fig. 2a and Table 1).

**Table 1:** Metadata of the sequencing data in this study. NA: not applicable.

Trio	Individual	Type	Tissue	Platform	Sequencing mode	Read N50 (bp)	Amount (Gb)	Read number (×10^6^)	Sequencing depth
Trio1	Female offspring	Genomic DNA	Muscle	PacBio Revio	HiFi	15,765	41.6	2.6	2.6
Trio1	Female offspring	Genomic DNA	Muscle	Illumina NovaSeq 6000	151-bp paired end	151	26.5	175.5	175.5
Trio1	Female offspring	Tissue mRNA	Muscle	Illumina NovaSeq 6000	101-bp paired end	101	7.3	71.9	71.9
Trio1	Female offspring	Tissue mRNA	Liver	Illumina NovaSeq 6000	101-bp paired end	101	6.2	61.4	61.4
Trio1	Sire	Genomic DNA	Muscle	Illumina NovaSeq 6000	151-bp paired end	151	28.8	190.7	190.7
Trio1	Dam	Genomic DNA	Muscle	Illumina NovaSeq 6000	151-bp paired end	151	27.7	183.7	183.7
Trio2	Male offspring	Genomic DNA	Muscle	PacBio Revio	HiFi	14,936	43.5	2.9	2.9
Trio2	Male offspring	Genomic DNA	Muscle	Illumina NovaSeq 6000	151-bp paired end	151	32.3	213.7	213.7
Trio2	Male offspring	Tissue mRNA	Muscle	Illumina NovaSeq 6000	101-bp paired end	101	9.2	90.7	90.7
Trio2	Male offspring	Tissue mRNA	Liver	Illumina NovaSeq 6000	101-bp paired end	101	6.6	65.5	65.5
Trio2	Sire	Genomic DNA	Muscle	Illumina NovaSeq 6000	151-bp paired end	151	25.4	168.4	168.4
Trio2	Dam	Genomic DNA	Muscle	Illumina NovaSeq 6000	151-bp paired end	151	29.5	195.2	195.2

### Genome assembly and quality metrics

Genome assembly and analysis were conducted as described in a protocol paper^26^. Parental Illumina DNA sequencing data were used to build *k*-mer hash tables of the two progenies using yak (version 0.1-r56; *yak count -b 37* for maternal and paternal Illumina sequencing data)^27^. HiFi reads of each progeny were assembled into contigs using the “Trio binning” mode of Hifiasm (version 0.19.5-r587; *hifiasm -1 paternal.yak -2 maternal.yak HiFi.fq.gz*)^23,24^. The resulting contigs were further scaffolded based on their sequence similarity with the chromosome-level Xu genome using RagTag (version v2.0.1; *ragtag.py scaffold*)^21,28^. Some short-read-based genome assemblies of *P*. *olivaceus* were available; however, they were too fragmented to be compared with our genome assemblies or the Xu genome. Consequently, we used only the Xu genome, as it exhibited the highest quality at the time of our analysis. Our four fully-phased chromosome-level genome assemblies exhibited comparable scaffold N50 lengths (26.13–26.68 Mb) to the previous Xu genome (26.05 Mb), in addition to larger genome assembly sizes^29–33^ (Fig. 2b and Table 2).

**Table 2:** Assembly summary statistics. NA: not applicable.

Assembly	Assembly size (bp)	Largest scaffold (bp)	Scaffold N50 (bp)	Assembly QV
Xu genome	588,170,514	31,859,138	26,045,262	NA
Trio1 offspring, maternal	612,885,529	31,024,304	26,474,190	53.52
Trio1 offspring, paternal	632,971,810	37,659,277	26,681,367	54.15
Trio2 offspring, maternal	634,549,395	38,489,622	26,324,942	53.28
Trio2 offspring, paternal	622,668,364	35,650,318	26,129,071	53.67

Assembly quality was evaluated by several computational methods. Quality values (QV) of HiFi-based assemblies were calculated based on independently generated short-read sequencing data using yak (version 0.1-r56; *yak count -b 37 -o short-read.yak* and *yak qv -p -l 100k sr.yak assembly.fasta*)^27^. Since no short-read sequencing data is available for the Xu genome, we could not evaluate its QV using yak. Our assemblies exhibited 53–54 adjusted QVs (99.9995%–99.9996% base-pair accuracy) (Table 2), implying their high base-level accuracy. Benchmarking Universal Single-Copy Orthologs (BUSCO) analysis was also conducted for the Xu genome and our genome assemblies (version 5.2.2; *busco -i input.fa -o output_prefix -m genome -l actinopterygii_odb10*)^29–35^. Our four assemblies showed comparable BUSCO completeness to the Xu genome (Fig. 2c).

Repetitive sequences of these genomes were identified using RepeatModeler (version 2.0.3; *RepeatModeler -LTRStruct -ninja_dir*) and masked using RepeatMasker (version 4.1.2-p1; *RepeatMasker -species metazoa -s* and *RepeatMasker -lib repeatmodeler_output-families.fa -s*)^36,37^. Composition of repetitive sequences were calculated based on hard-masking results.

The data revealed that repetitive elements were better resolved in our assemblies than the Xu genome, specifically for retroelements (50.17–58.08 Mb vs. 33.52 Mb) and small RNAs (2.31–3.18 Mb vs. 1.69 Mb) (Fig. 2d and Table 3). Our genome assemblies contained much longer repetitive sequences than the Xu genome, and these lengths largely explain the differences in genome size (genome size difference vs. repetitive sequence size difference: 25 Mb vs. 26 Mb for Trio1, maternal; 45 Mb vs. 35 Mb for Trio1, paternal; 46 Mb vs. 27 Mb for Trio2, maternal; and 34 Mb vs. 33 Mb for Trio2, paternal, respectively). This is because our genome assemblies also include small, highly repetitive contigs in addition to the chromosomes, while the publicly available Xu genome contains only scaffolded chromosomes. It is noteworthy that the genome sizes of flatfish species are smaller than those of other teleost species due to the significantly shorter repetitive and genic sequences^38^.

**Table 3:** Length and genomic composition of repetitive sequences.

Type	Xu genome	Trio1, maternal	Trio1, paternal	Trio2, maternal	Trio2, paternal
Retroelements (bp)	33,516,178	53,188,800	58,077,221	50,173,102	57,924,130
Genomic percentage	5.70	8.68	9.18	7.91	9.30
DNA transposons (bp)	32,148,478	35,527,015	37,201,530	35,646,232	35,821,153
Genomic percentage	5.47	5.80	5.88	5.62	5.75
Rolling-circles (bp)	4,811,911	4,652,616	5,045,882	4,921,233	4,811,713
Genomic percentage	0.82	0.76	0.80	0.78	0.77
Unclassified interspersed repeats (bp)	54,480,155	56,572,834	57,608,027	59,122,462	56,980,570
Genomic percentage	9.26	9.23	9.10	9.32	9.15
Small RNAs (bp)	1,688,225	2,529,378	2,310,791	2,742,188	3,181,860
Genomic percentage	0.29	0.41	0.37	0.43	0.51
Satellites (bp)	817,494	770,400	774,382	715,368	763,107
Genomic percentage	0.14	0.13	0.12	0.11	0.12
Simple repeats (bp)	15,971,040	16,669,898	17,088,762	17,278,262	16,786,290
Genomic percentage	2.72	2.72	2.7	2.72	2.7
Low complexity (bp)	3,193,610	3,180,542	3,312,082	3,399,776	3,275,752
Genomic percentage	0.54	0.52	0.52	0.54	0.53
Sum (bp)	146,627,115	173,091,511	181,418,705	173,998,650	179,544,603
Genomic percentage	24.93	28.24	28.66	27.42	28.83

**Fig. 3:**
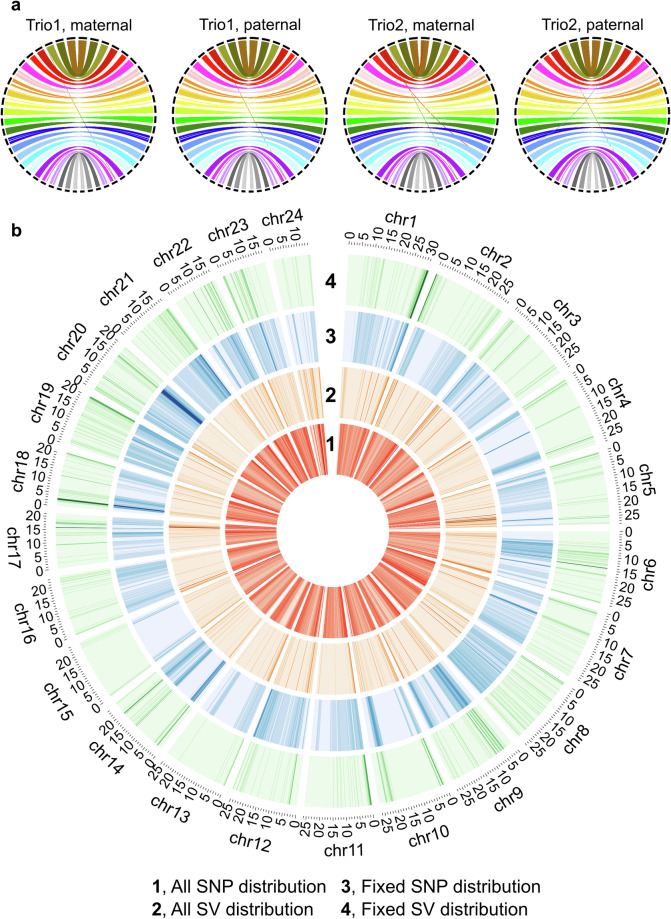
Genome-wide analysis of our genomes. (**a**) Synteny relationship between the Xu genome (left) and our four genome assemblies (right). Each coloured line represents a common BUSCO gene pair between the assemblies, with each colour denoting a chromosome. Black bars indicate chromosomes. (**b**) Genome-wide distributions of SNPs and SVs. Red and orange heatmaps represent all SNPs and SVs found in our genome assemblies compared to the Xu genome as a reference. Blue and green heatmaps represent fixed SNPs and SVs, only. Darker colours represent denser variants.

Additionally, we also analysed synteny between the Xu genome and our genome assemblies using common single-copy orthologs obtained from the BUSCO analysis. We found that almost all the genes were collinear across the chromosomes (Fig. 3a), supporting the scaffold-level accuracy of our genome assemblies. All these lines of evidence support that our genome assemblies are comparable to or even better than the previous chromosome-level assembly, Xu genome.

It is also significant that sex chromosomes have not yet been assigned in this species. In fish, sex determination can be driven by diverse mechanisms, such as sex chromosomes based on sex-determining genes or temperature-dependent gene activation^39–41^. *P*. *olivaceus* is known to have an XX/XY chromosome-based sex determination system^42,43^. Its gynogenetic offspring exhibit female-dominant phenotypes, supporting the XX/XY system^42^. The sex determination of *P*. *olivaceus* has been extensively analysed at both genomic and transcriptomic levels, with the *amh* gene suggested as a master sex-determining gene. Moreover, the *cyp11a* gene is known to play a crucial role in gonadal differentiation and development^44^. However, these data are still insufficient to conclusively determine which chromosome is the sex chromosome, and morphologically distinct sex chromosomes have not been clearly identified^45–49^. Thus, we were unable to assign sex chromosomes to our genome assemblies.

### Genome annotation for genes, pan-genome graph-based variants, and 5-methylcytosine sites

We further annotated our genome assemblies in terms of genes and graph-based pan-genome variants. To annotate genes in our genome assemblies, their corresponding RNA-seq reads were mapped to the soft-masked genome assemblies using HISAT2 (version 2.2.1; *hisat2-build index genome.fasta index* and *hisat2 -x index -1 read1.fastq -2 read2.fastq*), and the mapping results were sorted and indexed using SAMtools (version 1.11; *samtools sort* and *samtools index*)^50^. The sorted and indexed BAM files were used as evidence to annotate genes using BRAKER2 (version 2.1.6; *braker.pl --genome=genome.fasta --bam=hisat2_output.bam --softmasking*)^50–59^. A total of 30,968–32,526 protein-coding genes were annotated for our genome assemblies, whose N50 lengths of the coding sequence varied from 2,136–2,151 bp (Table 4).

**Table 4:** Summary statistics of annotated protein-coding genes.

Assembly	Number of protein-coding genes	N50 length (bp)	Homologous to any known proteins	Ratio (%)	Mean coverage (%)	Mean bit score	Homologous to RefSeq genes	Ratio (%)	Mean coverage (%)	Mean bit score	RNA-seq support (≥1 read)
Trio1 offspring, maternal	30,968	2,151	24,531	79.21	91.97	887	22,796	73.61	89.20	980	13,131
Trio1 offspring, paternal	32,115	2,142	25,686	79.98	92.15	883	23,883	74.37	89.58	979	13,520
Trio2 offspring, maternal	32,526	2,136	25,934	79.73	92.30	891	24,134	74.20	89.91	988	12,991
Trio2 offspring, paternal	31,176	2,145	24,791	79.52	91.94	887	22,916	73.51	89.34	985	12,673

We searched known homologous proteins of these *P*. *olivaceus* protein coding genes using MMseqs 2 (version 9cc89aa594131293b8bc2e7a121e2ed412f0b931; *mmseqs easy-search UniProtKB_database output_file_name tmp -s 7 --format-output "query,target,evalue,bits,qcov,theader,taxlineage"*)^60,61^. Among the ~30 K protein-coding genes, ~25 K genes (~80%) had homologous proteins in the UniProt Knowledgebase (Release 2023_03 that consists of UniProtKB/Swiss-Prot Release 2023_03 of 28-Jun-2023 and UniProtKB/TrEMBL Release 2023_03 of 28-Jun-2023)^62^ and ~23 K genes for the *P*. *olivaceus* protein sequences in the RefSeq database^10^ (Table 4). Their mean coverage and mean bit score were ~92% and ~890 for the UniProt database and ~89% and ~980 for the RefSeq database, implying their reliable homology (Table 4). We integrated gene information of the RefSeq database to our annotation files using AGAT (version 1.2.0; *agat_convert_sp_gxf2gxf.pl -g ours.gtf -o ours.gff*, *agat_sp_extract_attributes.pl -g RefSeq.gff -att ID,Dbxref,Name,gbkey,gene,product,gene_biotype,locus_tag -o RefSeq.attributes.txt*, and *agat_sq_add_attributes_from_tsv.pl --gff ours.gff --tsv RefSeq.attributes.txt -o ours.withRefSeq.gff*)^63^.

To assess which annotated genes have transcript-level evidence, RNA sequencing reads from muscle and liver tissues were mapped to each genome. Absolute and normalised read counts of each gene were calculated using the *summarizeOverlaps* function from the *GenomicAlignments* package (v1.38) and *edgeR* (v4.0.2)^64–66^. We found that about 13 K genes were supported by ≥1 RNA sequencing reads collected from muscle or liver tissues (Table 4). Since around 6 K genes were not supported by either protein homologs or RNA-seq evidence, it is possible that these genes may be expressed in other tissues or are false-positive genes, necessitating further validation.

Variants were called using the Minigraph-Cactus pipeline for pan-genome graph construction. We utilized the Xu genome as a reference and our four fully-phased genome assemblies as queries (version 2.6.4; *cactus-pangenome./jobstorepath./sequenceFile.tsv --outDir P.olivaceusPanGenome --outName P.olivaceus --reference Xu --vcf --giraffe --gfa --gbz*)^67,68^. This variant calling process annotated a total of fully-phased 6.64 M variants (Table 5). Because our four genome assemblies could be represented by up to four alternative alleles compared to the Xu genome, we counted the number of loci that have more than five alternative alleles. Out of the 6.64 M variant loci, only 118 loci (0.002%) did so, implying reliable variant calling. Among the variants, we aimed to identify fixed variants in the Korean population by analysing those that exhibited only a reference and a single alternative allele. Among the 5.01 M single-nucleotide polymorphisms (SNPs) and 0.08 M structural variants (SVs; variant size ≥ 50 bp), 4.99 M SNP loci (99.5%) and 30,744 SV loci (38.9%) had a single reference-type allele and a single alternative allele, only. Among these single-alternative-allele variants, 14.1% of SNPs and 13.4% of SVs were homozygous in both Trio1 and Trio2 genomes, which could be fixed during their genomic breeding process in Korea (Table 5).

**Table 5:** Summary statistics of genetic variants.

Type	At most four alternative alleles	Ratio (%)	Only a single alternative allele	Ratio (%)
Total	6,637,598	100.00	6,241,494	94.03
SNV	4,986,161	100.00	4,986,161	100.00
SV (≥50 bp)	100,956	99.99	31,495	31.19
**Type**	**No homozygous**	**Ratio (%)**	**Homozygous**	**Ratio (%)**
Total	4,013,836	64.31	2,227,658	35.69
SNV	3,158,386	63.34	1,827,775	36.66
SV (≥50 bp)	21,397	67.94	10,098	32.06
**Type**	**Fixed homozygous**	**Ratio (%)**	**Non-fixed**	**Ratio (%)**
Total	851,053	13.64	5,390,441	86.36
SNV	701,422	14.07	4,284,739	85.93
SV (≥50 bp)	4,255	13.51	27,240	86.49

These fixed variants were specifically enriched on the right arms of Chr1 and Chr20 and the left arm of Chr18 (Fig. 3b). Some other fixed loci on Chr3, Chr6, and Chr11 contained or were closely located to candidate genes associated with growth traits and virus resistance, such as *nlgn1*, *plcg1*, and various immune-response genes^3,69^. Since the two Korean olive flounders have been selectively bred for their improvement, these fixed loci may contain important breeding genes and alleles, which should be further analysed for future genomic selection. It is important to note that our genome assemblies represent only four haplotypes, and thus they cover a limited portion of the *P*. *olivaceus* population in Korea. In the near future, we hope that many more cultured and wild individuals will be sequenced at the chromosome level to create a more comprehensive pangenome reference for *P*. *olivaceus*.

We assessed 5-methylcytosine (5mC) base information that can be automatically detected by the PacBio Revio system. We mapped HiFi reads in a BAM-formatted file to the Xu genome using pbmm2 (version v1.12.0; *pbmm2 align XuGenome.fa sample_HiFi.bam output.bam --preset HIFI --sort*)^70^, and the output mapping file was processed by pb-CpG-tools (version 2.3.1; *aligned_bam _to_cpg_scores --bam output.bam --output-prefix output --model pileup_calling_model.v1.tflite*)^71^. We identified ~10.9 M 5mC sites for each sample and filtered them using the 90% methylation probability cutoff, resulting in 3.4–3.7 Mb potential methylated sites (Table 6). These filtered methylated sites covered ~0.6% of the genome or ~16% of its CpG sites.

**Table 6:** Potential 5-methylcytosine sites.

Individual	All potential 5mC sites	5mC sites, ≥90% probability	Ratio to the genome size (%)	Ratio to the CpG sites (%)
Trio1 offspring	10,916,263	3,706,588	0.63	16.68
Trio2 offspring	10,887,242	3,427,028	0.58	15.42

### Synteny analysis using other flatfish genomes

**Fig. 4:**
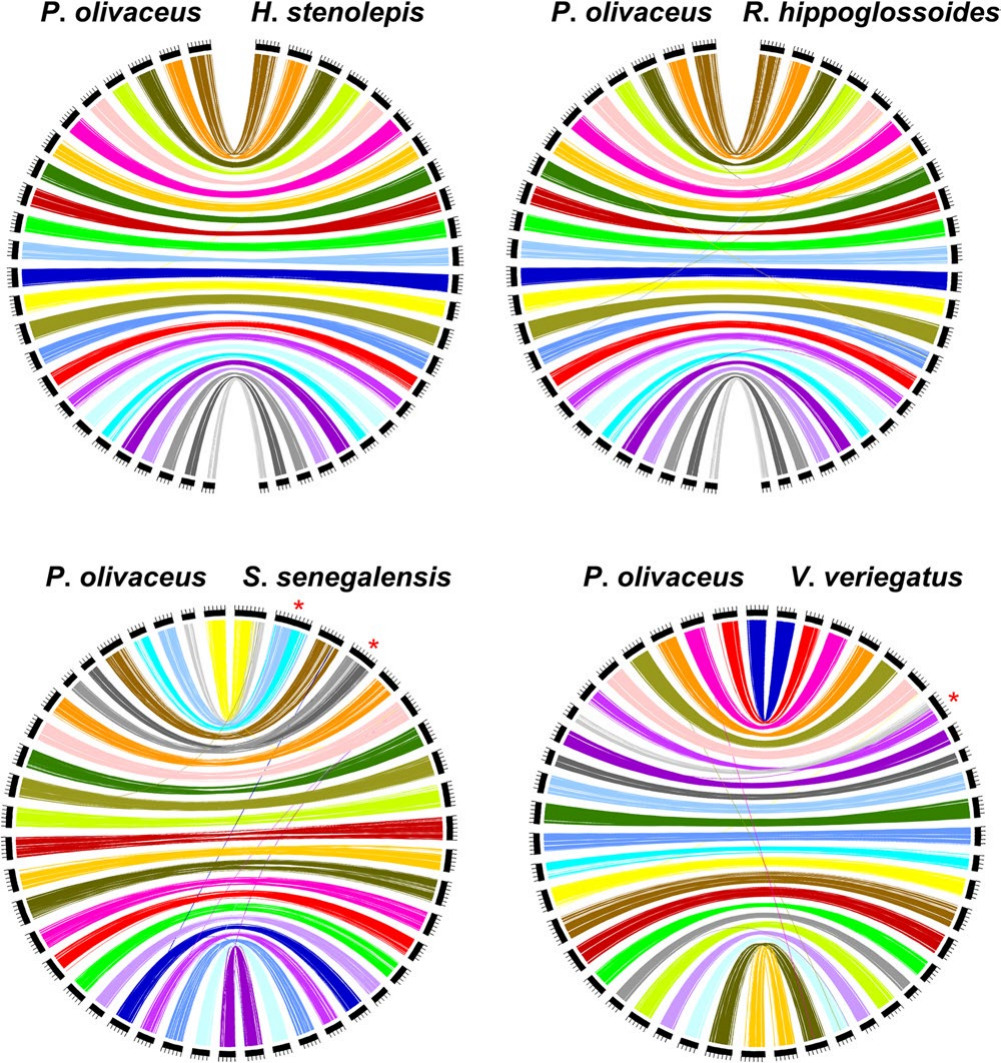
Synteny relationships between *Paralichthys olivaceus* and the close relative species in the family, Pleuronectiformes (*Hippoglossus stenolepis*, *Reinhardtius hippoglossoides*, *Solea senegalensis*, and *Verasper variegatus*). Asterisks represent fusion chromosomes. Each coloured line represents the common BUSCO gene pair. The same colour represents the same chromosome of *P*. *olivaceus*.

It is well known that *Solea senegalensis* (haploid karyotype, n = 21) and *Verasper variegatus* (n = 23) have fewer chromosomes than other flatfish (n = 24) and that these fewer chromosomes may have resulted from Robertsonian fusion events^72–74^. To support these data, we also analysed synteny among chromosome-level flatfish genome assemblies (*Hippoglossus stenolepis*, *Reinhardtius hippoglossoides*, *Solea senegalensis*, and *Verasper variegatus*) by comparing their chromosomes with our *P*. *olivaceus* chromosomes (Trio 1, maternal assembly)^75–77^. Common complete single-copy orthologs identified by our BUSCO analysis were utilized for this synteny analysis, and we visualized the data using Circos (version circos | v 0.69-8 | 15 Jun 2019 | Perl 5.026002)^78^. Our synteny analysis revealed that almost all chromosomes in these flatfish were collinear with our *P*. *olivaceus* chromosomes, except for chromosomes 1 and 2 in *S*. *senegalensis* and chromosome 7 in *V*. *variegatus* (Fig. 4). These exceptional chromosomes exhibited the signature of Robertsonian fusion events, as each exceptional chromosome showed collinear relationships with two different chromosomes in *P*. *olivaceus*.

## Data Records

All sequencing reads and assembly from this study have been deposited in the NCBI BioProject database (https://www.ncbi.nlm.nih.gov/bioproject) under the accession numbers PRJNA1052749, PRJNA1128064, and PRJNA1128063. The raw reads are available at NCBI SRA^25^. The four fully-phased genome assemblies are available at NCBI GenBank^30–33^. Genome sequences and their annotation files are also available at figshare (10.6084/m9.figshare.27021649)^59^. The following files contain long-read-based genome assembly sequences (FASTA-formatted) and responsible gene annotations (GFF-formatted): ‘Trio1_Offspring.maternal.scaffold.fa.gz’ and ‘Trio1_Offspring.maternal.scaffold.AGAT.withRefSeq_GCF_001970005.1.gff.gz’ for the Trio1, maternal genome assembly; ‘Trio1_Offspring.paternal.scaffold.fa.gz’ and ‘Trio1_Offspring.paternal.scaffold.AGAT.withRefSeq_GCF_001970005.1.gff.gz’ for Trio1, paternal genome assembly; ‘Trio2_Offspring.maternal.scaffold.fa.gz’ and ‘Trio2_Offspring.maternal.scaffold.AGAT.withRefSeq_GCF_001970005.1.gff.gz’ for Trio2, maternal genome assembly; and Trio2_Offspring.paternal.scaffold.fa.gz and ‘Trio2_Offspring.paternal.scaffold.AGAT.withRefSeq_GCF_001970005.1.gff.gz’ for Trio2, paternal genome assembly. An output of pangenome analysis (VCF-formatted) is contained in the file, ‘P.olivaceus.vcf.gz’. Methylcytosine information is available for ‘Trio1_Offspring_to_XuGenome.bed.gz’ for Trio1 offspring and ‘Trio2_Offspring_to_XuGenome.bed.gz’ for Trio2 offspring (BED-formatted; Each column represents reference chromosome name, start position, end position, methylation score, haplotype, coverage, estimated count of methylation, estimated count of non-methylation, and modification probability, respectively).

## Technical Validation

Assembly quality was assessed using contig length, quality values, and BUSCO. N50 lengths of the four assemblies were longer than 15 Mb, and quality values were ~53 (~99.9995% base-pair accuracy). Their complete BUSCO values exceeded 96%.

## Data Availability

All scripts, parameters, and options are described in the method section. We used publicly available programs and do not use custom programs.
