# Author Correction: Fully phased genome assemblies and graph-based genetic variants of the olive flounder, Paralichthys olivaceus

**DOI:** 10.1038/s41597-024-04273-1

**Published:** 2024-12-20

**Authors:** Julan Kim, Yoonsik Kim, Jeongwoen Shin, Yeong-Kuk Kim, Doo Ho Lee, Jong-Won Park, Dain Lee, Hyun-Chul Kim, Jeong-Ho Lee, Seung Hwan Lee, Jun Kim

**Affiliations:** 1 Genetics and Breeding Research Center, National Institute of Fisheries Science, Geoje, 53334 Korea; 2 Department of Bio-AI Convergence, Chungnam National University, Daejeon, 34134 Republic of Korea; 3 Quantomic research and solution, Yuseong-gu Daejeon Tips-town, Daejeon, 34134 Korea; 4 Division of Animal & Dairy Science, Chungnam National University, Daejeon, 34134 Korea; 5 Research and Development Planning and Coordination Department, National Institute of Fisheries Science, Busan, 46083 Korea; 6 Department of Convergent Bioscience and Informatics, Chungnam National University, Daejeon, 34134 Korea

**Keywords:** Animal breeding, DNA sequencing, Comparative genomics, Structural variation, Animal breeding, DNA sequencing, Comparative genomics, Structural variation

Correction to: *Scientific Data* 10.1038/s41597-024-04033-1, published online 4 November 2024

In the article the affiliation ‘Bigdata Center, TNT Research Co., Lts, Wonjangdong-gil 102, Deokjin-gu, Jeollabuk-do, Jeonju-si, 54810, Korea’ was inadvertently left in the affiliation list. All affiliations for the authors have been updated. Second, the authors omitted the following statement ‘7. These authors contributed equally: Julan Kim, Yoonsik Kim’. The original article has been corrected.

